# Temperature and Pressure Dependences of the Elastic Properties of Tantalum Single Crystals Under <100> Tensile Loading: A Molecular Dynamics Study

**DOI:** 10.1186/s11671-018-2526-1

**Published:** 2018-04-24

**Authors:** Wei-bing Li, Kang Li, Kang-qi Fan, Da-xing Zhang, Wei-dong Wang

**Affiliations:** 1 ZNDY of Ministerial Key Laboratory, Nanjing University of Science and Technology, Nanjing, 210094 People’s Republic of China; 2 McCormick School of Engineering and Applied Science, Northwestern University, Evanston, IL 60208 USA; 3 School of Mechano-Electronic Engineering, Xidian University, Xi’an, 710071 People’s Republic of China; 4 Research Center of Micro-Nano Systems, Xidian University, Xi’an, 710071 People’s Republic of China

**Keywords:** Tantalum single crystals, Elastic properties, Pressure dependence, Temperature dependence, MD simulations, Tensile loading

## Abstract

Atomistic simulations are capable of providing insights into physical mechanisms responsible for mechanical properties of the transition metal of Tantalum (Ta). By using molecular dynamics (MD) method, temperature and pressure dependences of the elastic properties of Ta single crystals are investigated through <100> tensile loading. First of all, a comparative study between two types of embedded-atom method (EAM) potentials is made in term of the elastic properties of Ta single crystals. The results show that Ravelo-EAM (Physical Review B, 2013, 88: 134101) potential behaves well at different hydrostatic pressures. Then, the MD simulation results based on the Ravelo-EAM potential show that Ta will experience a body-centered-cubic (BCC) to face-centered-cubic (FCC) phase transition before fracture under <100> tensile loading at 1 K temperature, and model size and strain rate have no obvious effects on tensile behaviors of Ta. Next, from the simulation results at the system temperature from 1 to 1500 K, it can be derived that the elastic modulus of *E*_100_ linearly decrease with the increasing temperature, while the yielding stress decrease with conforming a quadratic polynomial formula. Finally, the pressure dependence of the elastic properties is performed from 0 to 140 GPa and the observations show that the elastic modulus increases with the increasing pressure overall.

## Background

In general, Tantalum (Ta) belongs to BCC structure at ambient condition. At present, numerous literatures have proved that Ta single crystals show excellent phase stability [1–3] under high pressures. What is more, Ta has a very high melting temperature 3269 K at ambient pressure which is higher than most of other metals [4]. Because of its excellent properties, Ta is an ideal material for many technological applications, such as diffusion barrier in micro/nanoelectronics, wear protection coating, and high-temperature super-alloys.

Recently, a great deal of effort both in experimental [2, 5–7] and theoretical [8–14] fields have been put into the high-pressure and high temperature properties of Ta. Dewaele et al. [5] studied the effects of pressure on the yield strength of Ta in a diamond-anvil cell (DAC) up to 93 GPa, and the DAC experiments also presented the BCC structure remained stable up to 135 GPa [2]. In addition, Shigeaki [8] simulated equation of state (EOS) of Ta up to 100 GPa and 3000 K using DFT. Wu et al. [9] investigated the elastic and thermodynamic properties of Ta at high-pressures up to 350 GPa. Meanwhile, Škoro et al. [6, 7] measured the yield strength and Young’s modulus of Ta at very high temperatures up to 2250 and 2500 K, respectively. Gu et al. [10] performed a study on the high-pressure structure and elastic properties of the cubic Ta up to 500 GPa using the first-principles method. It is found that the elastic constants as a function of pressure, and that bulk, Young’s, and shear modulus of Ta all increased with the increasing pressure.

In addition to DAC experiments and DFT calculations, there are also many studies at high temperature and high pressure in the field of MD simulations [15–18]. Liu et al. [15] used the extended Finnis-Sinclair (EFS) potential and investigated thermal EOS as well as melting properties of Ta at pressures up to 400 GPa. Besides, Tramontina et al. [16, 17] studied the effects of crystal orientation on plasticity mechanisms at high pressures. They also discussed the influence of shock strength and shock rise time on their microstructures. Furthermore, Ruestes et al. [18] carried out indentation simulations for BCC Ta using three different interatomic potentials and presented the defect mechanisms responsible for the creation and expansion of the plastic deformation zone.

Despite the numerous investigations above, there has not been a systematic atomistic simulation study of the dynamic response of Ta under tensile loading using MD simulations. The main purpose of the present work is to investigate the elastic properties of Ta single crystals under <100> tensile loading, considering the effects of size, strain rate, temperature, and pressure. In addition, to understand whether a phase transition could be induced through <100> tensile loading is another purpose for this work.

## Methods/Experimental

### Physical Modeling

**Fig. 1:**
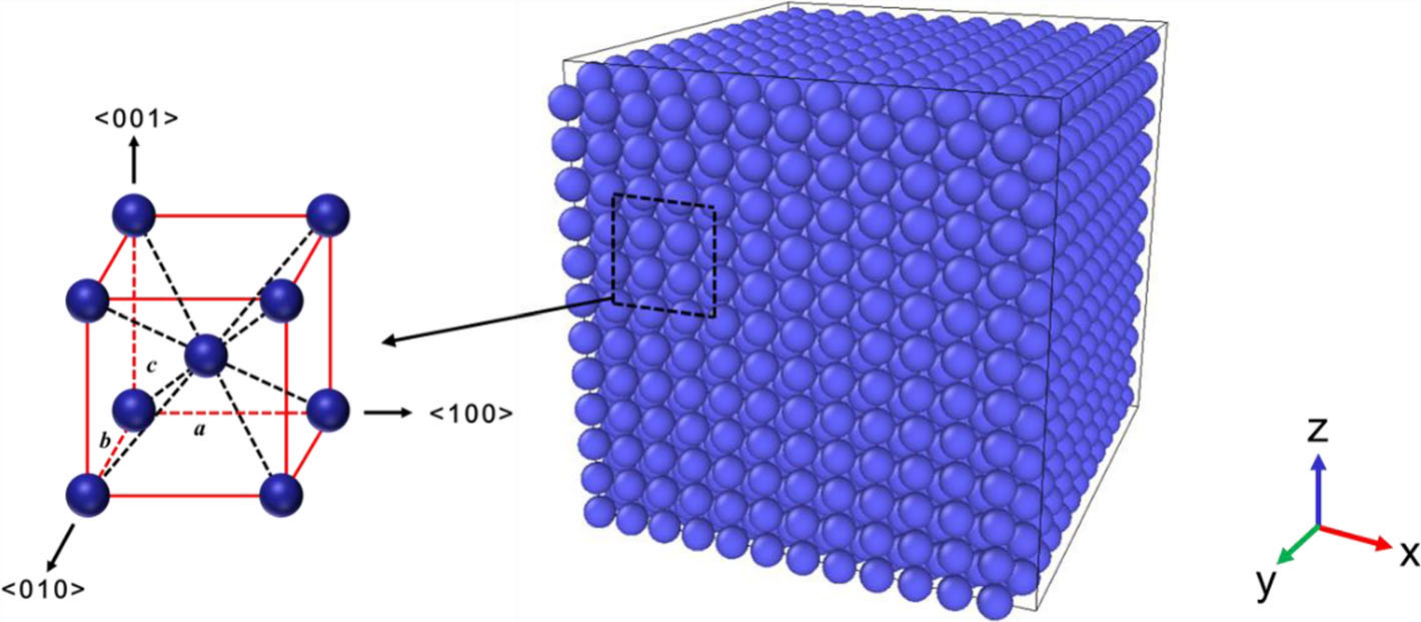
BCC structure and sketch map of the Ta cubic with the edge length of 3.96 nm. The sketch map is the original structure in our present simulations

As shown in Fig. 1, the Ta cubic investigated in this paper are generated by repeating a BCC unit cell along the <001>, <010>, and <100> orientations and the lattice parameters are *a* = *b* = *c* = 3.301 Å, respectively. Four cubic models with different edge lengths, including 12*a* (3.96 nm), 18*a* (5.94 nm), 24*a* (7.92 nm), and 30*a* (9.90 nm), are constructed. The corresponding number of atoms is 3456, 11,664, 27,648, and 54,000, respectively. Figure 1 shows the sketch map of Ta cubic with the edge length of 3.96 nm, which is the original structure in our present simulations.

### MD Simulation Details

Our MD simulations reported in this paper are performed using the large-scale atomic/molecular massively parallel simulator (LAMMPS) [19]. During the process of MD simulation, the external force applied on Ta atoms is calculated according to the interatomic potential functions between those atoms. In this study, two different interatomic potentials are considered: the EAM potential by Zhou et al. [20] and Ravelo et al. [21], respectively. Both potentials were applied in reproducing some elastic properties of Ta which are listed in Table 1. For the sake of simplicity, they will be referred as Ravelo-EAM and Zhou-EAM potentials.

**Table 1:** Elastic properties of Ta at 0 and 0 GPa by the two EAM potentials of Zhou-EAM and Ravelo-EAM, together with experimental values by Stewart et al. [34]

	*C*_11_(GPa)	*C*_12_(GPa)	*C*_44_(GPa)	*E*_100_(GPa)	*E*(GPa)
Ravelo-EAM	262.6	160.7	81.8	140.5	181.9
Zhou-EAM	264.4	159.5	80.9	144.3	182.6
Experiments	264.0	160.0	82.0	142.6	183.5

The elastic behavior of structures with cubic symmetry is described completely by their elastic constants, *C*_11_, *C*_12_, and *C*_44_. The orientation-dependent elastic modulus for <100>, <110>, and <111> single crystals are calculated by means of the several equations [18]. In our work, MD simulations are performed to study the effects on the elastic modulus under <100> tensile loading. Hence, we focus on the elastic modulus for <100 > orientation and take the elastic modulus of *E*_100_ into account. Consequently, we consider the elastic constants *C*_11_ and *C*_12_ as well as the following equation [22]:

1 $$ {E}_{100}=\frac{\left({C}_{11}-{C}_{12}\right)\left({C}_{11}+2{C}_{12}\right)}{\left({C}_{11}+{C}_{12}\right)} $$

**Fig. 2:**
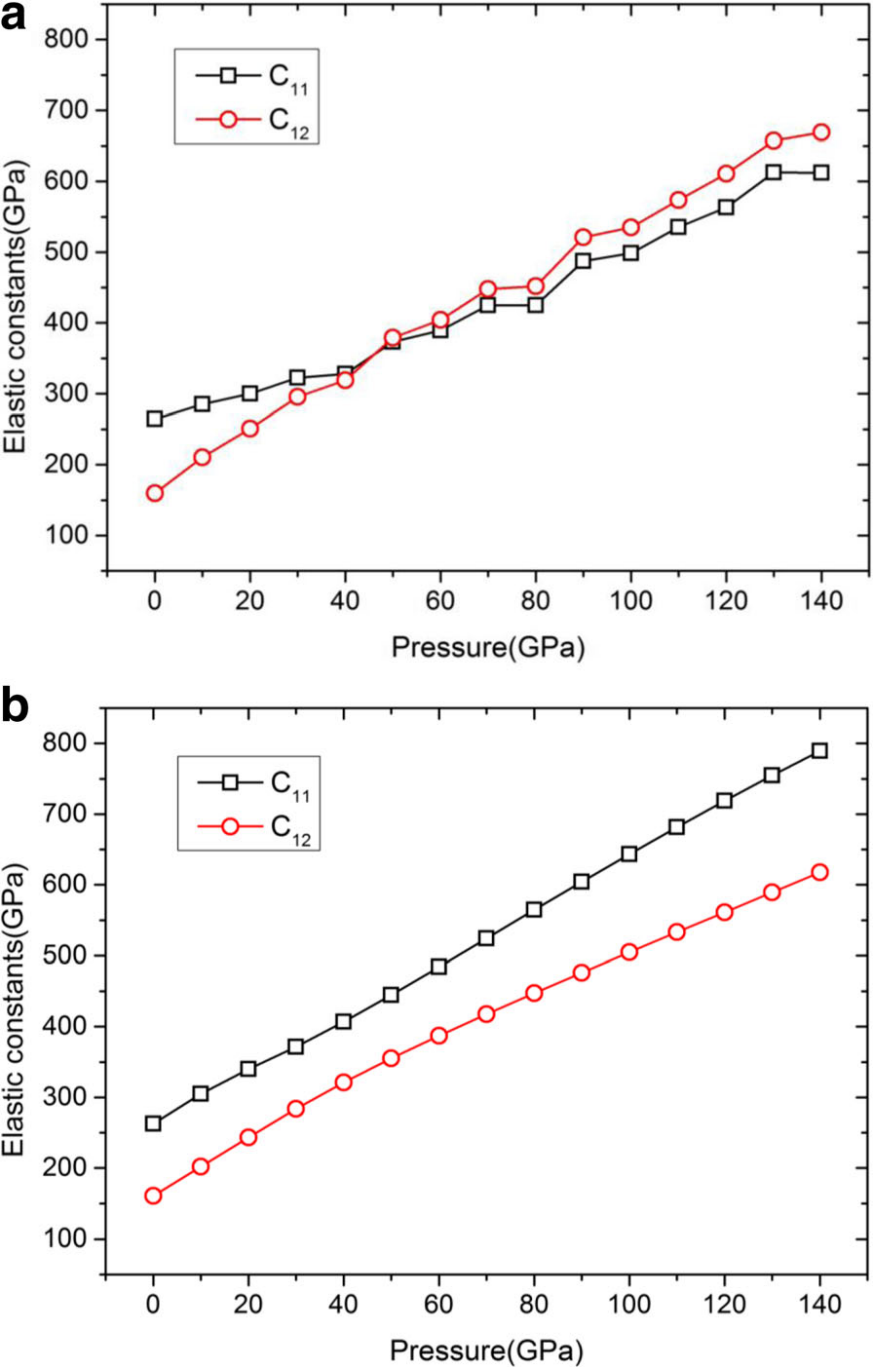
Pressure dependence of the elastic constants of *C*_11_ and *C*_12_ based on the potentials studied. **a** Zhou-EAM. **b** Ravelo-EAM. In present work, we utilized the direct method to calculate the elastic constants *C*_11_ and *C*_12_ for two types of EAM potentials. The C11/C12-pressure curves for two potentials are shown in **a** and **b**, respectively

In general, there are three methods to calculate the elastic constants from MD including stress-fluctuation method, strain-fluctuation method, and direct method. In present work, the direct method which is similar to that of Gao et al. [23] is utilized to calculate the elastic constants *C*_11_ and *C*_12_ for two types of EAM potentials, as shown in Fig. 2.

From Fig. 2a, one can find that the obtained curves of both *C*_11_ and *C*_12_ based on the Zhou-EAM potential cannot keep smooth-going with the increasing pressure up to 140 GPa. There is a cross-over point at the pressure of ~ 40 GPa, i.e., *C*_11_ = *C*_12_, at which the elastic modulus of *E*_100_ will be zero according to Eq. (1). Moreover, *E*_100_ will present a negative value the pressure higher than 40 GPa, which is suspicious and conflict with the theoretical and experimental results [24, 25]. Therefore, the Zhou-EAM potential performs poorly in the range considered here. Then, let us examine the feasibility of the Ravelo-EAM potential from the MD simulation results of *C*_11_ and *C*_12_ based on the Ravelo-EAM potential pictured in Fig. 2b. Numerical results show that the higher the pressure, the bigger the values of both *C*_11_ and *C*_12_, which agrees well with the trend of the elastic constants changing with pressures through DFT calculations [9, 25]. Meanwhile, the results calculated using the Ravelo-EAM potential is remarkable the same as the values reported by Ruestes et al. [18]. The Ravelo-EAM potential behaves well under high pressure and at the same time it can also describe the elastic and mechanical properties of Ta under dynamic deformation [26]. Therefore, we will perform our simulations based on the Ravelo-EAM potential in the following sections.

After geometric construction, we carry out a series of relevant MD simulations. During the MD simulations, the periodic boundary conditions (PBC) are used in all three directions of the cubic models. The time step is set as 1 fs, and the system temperature is set as 1, 300, 600, 900, 1200, and 1500 K to explore the temperature dependence of elastic properties of Ta. First, the model is relaxed about 50 ps relaxation process using a canonical ensemble (NVT) MD simulation in order that the system lies in local potential minimum. Then, it used isothermal-isobaric (NPT) MD simulation to ensure a specified hydrostatic pressure ranging from 0 to 140 GPa to study the effects of pressure on elastic properties of Ta [27]. Finally, a tensile loading with a strain rate ranging from 5 × 10^8^ s^− 1^ to 7.5 × 10^9^ s^− 1^ [28, 29] is applied in x-direction of Ta cubic. Meanwhile, NPT simulation is performed in the y- and z-directions at the same pressure applied in the second step. Therefore, the elastic modulus calculated here is for <100> orientation. For all MD simulations, the models will be stretched to an elongation of 15% in the x-direction through <100> tensile loading.

## Results and Discussion

### Stretching Process

**Fig. 3:**
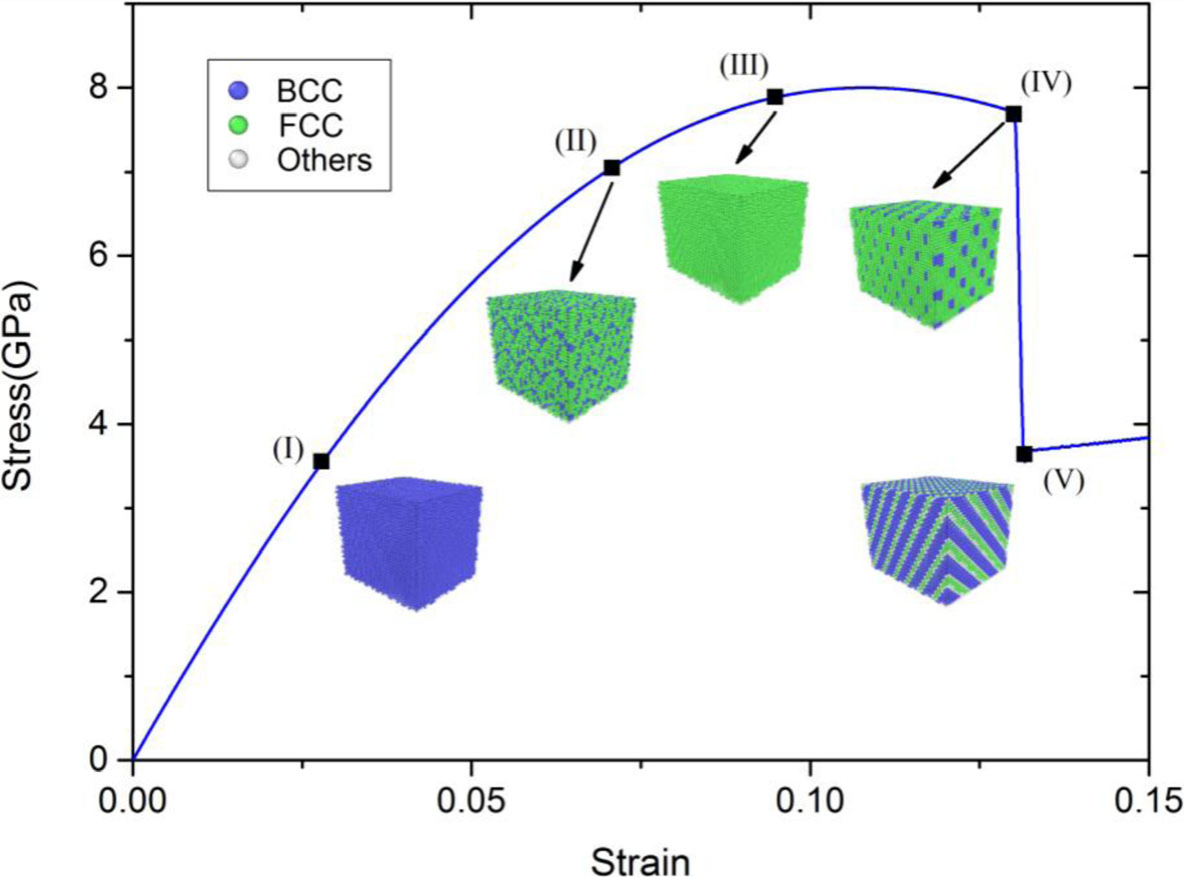
Stress-strain curve of Ta at zero pressure and the corresponding configurations with different strains. Notes: blue, green, and white balls are corresponding to BCC, FCC, and other lattice structures, respectively

During the stretching process, the simulated configurations are visualized using the scientific software package Open Visualization Tool (OVITO) [30]. The stress-strain curve of Ta under <100> uniaxial tensile strains at zero pressure and the corresponding atomic configurations with different strains are shown in Fig. 3.

As shown in Fig. 3, it can be concluded from the stress-strain curve that the surface fractures nearby the configuration (IV). At the beginning of the uniaxial tensile strains, the stress changes typically linearly with the strain and Fig. 3I shows that the atomic configurations maintain BCC structures. As the strain increases, the phase transition from BCC to FCC structures starts at the strain of ~ 7.4% and completes at the strain of ~ 9.8%, as shown in Fig. 3II, II, respectively. And these FCC structures keep the maximum before the first surface fracture. When the strain is ~ 13.1%, the edge lengths of y- and z-directions decrease abruptly, which results in surface fracture. Meanwhile, it is noteworthy that clusters happen at a very short time, as shown in Fig. 3IV. Under continuous uniaxial deformation, the atomic configuration maintain stripe-like until the strain is ~ 13.3%, which is shown in Fig. 3V.

In this paper, we concentrate on the dependence of tensile properties on model size, strain rate, temperature, and pressure, as discussed in this section. Theoretically, the model is stretched linearly in the elastic deformation stage and the elastic modulus is defined as the slope of the linear portion of the stress-strain curve. It can be found that all the models have the similar stretching process, and the stress-strain curves have the similar variation tendencies. Therefore, we use the same approach to gain the elastic modulus of Ta of various model sizes and strain rates.

### Dependence on Size and Strain Rate

Table 2 lists the elastic modulus and the yielding stress of the different model sizes at the temperature of 1 K and the strain rate of 5 × 10^8^ s^− 1^. It can be easily concluded that model sizes have no effects on elastic modulus and yielding stress of Ta. This is very easy to explain that the elastic modulus is to describe the interaction between atoms, while the elastic modulus does not vary with the model sizes. It can be seen from the Table 2 that the elastic modulus is ~ 139 GPa, remarkably the same as the simulation result of 140 GPa [18].

**Table 2:** Elastic modulus and yielding stress of different model sizes at 1 K and strain rate of 5 × 10^8^ s^− 1^

Sizes(nm)	Elastic modulus(GPa)	Yielding stress(GPa)
3.96	139.32	8.02
5.94	139.38	7.97
7.92	139.42	8.08
9.90	139.39	7.99

According to the existing Refs. [28, 29], most of strain rates range from 10^8^ s^− 1^ to 10^10^ s^− 1^. In this paper, four strain rates are selected to perform tensile simulations, including 5.0 × 10^8^ s^− 1^, 7.5 × 10^8^ s^− 1^, 5.0 × 10^9^ s^− 1^, and 7.5 × 10^9^ s^− 1^. Table 3 lists the elastic modulus and the yielding stress at the temperature of 300 K and at different strain rates. It can be easily concluded that strain rate has no obvious effects on the elastic modulus and the yielding stress.

**Table 3:** Elastic modulus and yielding stress of Ta at 300 K and different strain rates

Strain rates (s^−1^)	Elastic modulus (GPa)	Yielding stress (GPa)
5.0 × 10^8^	125.93	6.93
7.5 × 10^8^	125.30	6.86
5.0 × 10^9^	125.79	6.91
7.5 × 10^9^	125.14	6.96

Meanwhile, we also simulate the effects of model size and strain rate on the elastic modulus and the yielding stress under different temperatures and pressures. These simulations come to the same conclusion. Therefore, we will use the same model size of 3.96 nm and the same strain rate of 5 × 10^8^ s^− 1^ for the following simulations.

### Dependence on Temperature

**Fig. 4:**
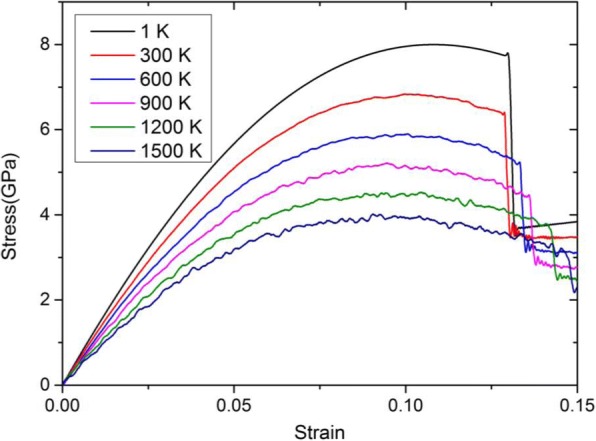
Stress-strain curves of 3.96 nm Ta model at the strain rate of 5 × 10^8^ s^− 1^ and different temperatures from 1 to 1500 K. It can be seen from Fig. 4 that the slopes of these curves that denotes the elastic modulus in <100> orientation, i.e., *E*_100_, during elastic tension period and the yielding stress are gradually decreasing with the increasing temperature

Figure 4 shows the stress-strain curves at different temperatures up to 1500 K. It can be seen that the slopes of these curves that denotes the elastic modulus in <100> orientation, i.e., *E*_100_, during elastic tension period and the yielding stress are gradually decreasing with the increasing temperature. According to the theory of thermodynamics [31], the total kinetic energy of all the atoms of the system generally satisfies the following equation:

2 $$ {E}_{\mathrm{k}}=\sum \limits_{i=1}^N\frac{1}{2}{mv_i}^2=\frac{3}{2}{Nk}_BT $$

where *E*_k_ is the total kinetic energy of the system; *N* is the total number of atoms; *K*_B_ is the Boltzmann constant; *T* is the thermodynamic temperature. Therefore, it can be concluded that the system contains greater total kinetic energy with the higher temperature, and the atoms move faster. From the thermodynamic point of view, the atoms become more active and the motion of the atoms is more intensive, which means greater amplitude in its equilibrium position. In stretching process, the attractive force between atoms relatively reduced and atoms escape from the equilibrium position easily, so the stress in x-direction reduces at the same strain. Therefore, the elastic modulus at higher temperature will be less than those at lower temperature. In addition, the trend of these curves agrees well with early theoretical and experimental findings of Ta [6, 7, 32].

In order to facilitate observation, Table 4 lists the elastic modulus and the yielding stress of Ta at different temperatures. The elastic modulus will decrease by ~ 42.3% from 136.49 to 76.67 GPa, and the yielding stress decrease by ~ 51% from ~ 8 to ~ 4 GPa as temperature increases from 1 to 1500 K.

**Table 4:** Elastic modulus and yielding stress at different temperatures

Temperature (K)	Elastic modulus (GPa)	Yielding stress (GPa)
1	136.49	8.02
300	126.95	6.86
600	114.82	5.91
900	101.28	5.18
1200	87.93	4.48
1500	76.67	3.92

**Fig. 5:**
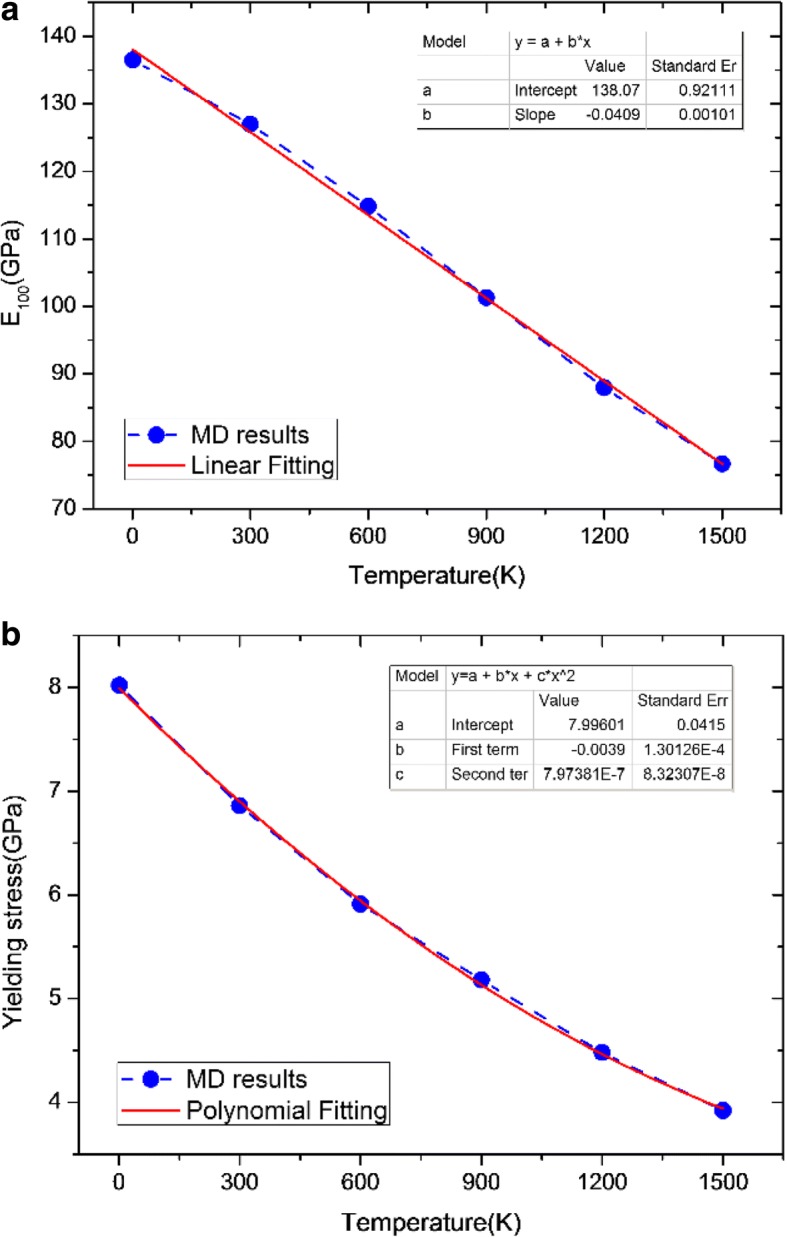
**a** Elastic modulus and **b** yielding stress versus temperature for Ta. In Fig. 5, we also present a parametrization of temperature dependence of the elastic modulus (*E*_100_) results

According to Table 4, we can further derive a parametrization of temperature dependence of the elastic modulus (*E*_100_) results shown as solid line in Fig. 5a. The parametrization reads as follows:

3 $$ {E}_{100}=a+{b}^{\ast }T $$

where *E*_100_ is in (GPa) and *T* is given in (*K*); *a* = 138.07 ± 0.92111, and *b* = − 0.04094 ± 0.00101. This equation shows that *E*_100_ linearly decrease with the increasing temperature, and it is recommended for use up to the temperature from 0 to 1500 K. From Eq. (3), it can be easily obtained that *E*_100_ will reach 0 GPa at the temperature of *T*_critical_ = −*a*/*b* = 3372 K which is very close to the melting temperature of Ta [15].

For the yielding stress, the recommended parametrization is

4 $$ {Y}_{\mathrm{stress}}=a+{b}^{\ast }T+{c}^{\ast }{T}^2 $$

where *Y*_stress_ is in (GPa) and *T* is given in (*K*); *a* = 7.99610 ± 0.0415, *b* = − 0.0039 ± 1.30126 × 10^− 4^, and *c* = 7.97381 × 10^− 7^ ± 8.32307 × 10^− 8^. From Eq. (4), it can be found that the yielding stress likely decrease with the temperature conforming a quadratic polynomial model, as given to solid line in Fig. 5b.

### Dependence on Pressure

As mentioned in the “Introduction” section, extensive theoretical and experimental efforts have been made on thermoelastic properties of Ta under high pressure conditions. Different from the static methods, we adopt a dynamic method through <100> tensile loading to examine its pressure dependence of the elastic modulus of *E*_100_ under different hydrostatic pressure.

**Fig. 6:**
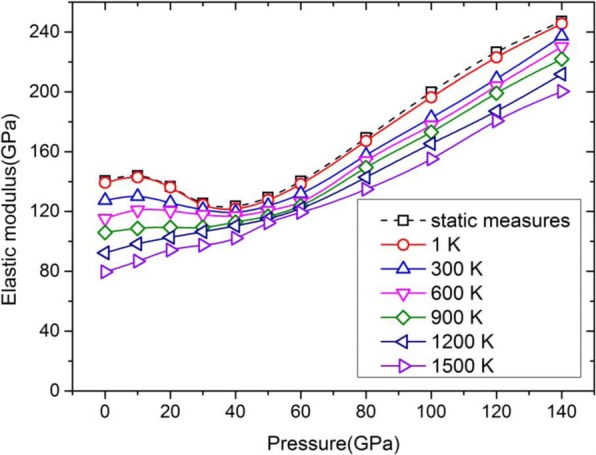
Elastic modulus in <100> orientation of Ta at different temperatures and pressures. It is worth pointing out that the elastic modulus of these curves is *E*_100_

Figure 6 shows the curves of the elastic modulus of *E*_100_ versus the pressure up to 140 GPa at different temperatures from 1 to 1500 K. While all solid lines with different colors are obtained through a dynamic method at different temperatures, the dotted line with square markers is obtained through a static method by using Eq. (1) based on the values of *C*_11_ and *C*_12_ at 0 K. It is obvious that the curve at 1 K, i.e., the red solid line with circle markers, is almost overlapped with the curve obtained through a static method (the dotted line), which denotes that the dynamic method adopted in present work is applicable at high pressure up to 140 GPa.

As illustrated in Fig. 6, the elastic modulus of *E*_100_ at a temperature no higher than 600 K shows a downward concave section as the pressure increases from 20 to 60 GPa. Ruestes et al. [18] reported the elastic constants as a function of pressure up to 60 GPa by using MD simulations, and the results agree well with the calculated *C*_11_ and *C*_12_ in present work. In return, the calculated elastic modulus of *E*_100_ also shows the same trend with our results. But the calculated elastic modulus of *E*_100_ from DFT calculations [33] gradually increases with the increasing pressure, and no downward concave section is found in the curves. What makes this inconsistency happen? In general, the potentials used in MD simulations are obtained by fitting DFT calculations and experimental results. In this sense, DFT calculations have higher accuracy than MD method. The Ravelo-EAM potential [21] is constructed by introducing high-pressure properties into the fitting DFT equation of state (EOS) curve of Ta single crystals. During fitting procedure, the cold curve of EOS is extended to include higher-order (cubic and quartic) terms of lattice constant, which makes MD EOS very sensitive to high-order terms of lattice constant. In other words, this inconsistency may be due to that the Ravelo-EAM potential could not precisely describe the EOS of Ta under the pressure from 20 to 60 GPa. On the whole, it can be concluded that the curves of elastic modulus versus pressure have a similar trend at different temperature, and the elastic modulus gradually increases with the increasing pressure over ~ 40 GPa.

## Conclusions

In this paper, a MD simulation has been performed to investigate temperature and pressure dependences of the elastic properties of Ta single crystals through <100> tensile loading. At first, we made a comparative study on two types of EAM potentials, including Zhou-EAM and Ravelo-EAM, in terms of the elastic properties of Ta at 0 K and different hydrostatic pressures. The results show that the Ravelo-EAM potential behaves better than Zhou-EAM potential under different pressures. Then, MD simulations on tensile behaviors of Ta single crystals are carried out based on the Ravelo-EAM potential. The observations show that Ta will experience a BCC-FCC phase transition before fracture under <100> tensile loading, and the model size and strain rate have no obvious influence on tensile behaviors of Ta single crystals. Moreover, the elastic modulus of *E*_100_ will linearly decrease from ~ 136 to ~ 79 GPa with the increasing temperature from 1 to 1500 K, and the yielding stress decrease from ~ 8 to ~ 4 GPa with the increasing temperature, conforming a quadratic polynomial formula. Finally, the pressure dependence of the elastic properties is performed from 0 to 140 GPa, and the observations show that the elastic modulus increases with the increasing pressure overall. The results from MD simulations also show that the Ravelo-EAM potential behaves well at higher pressure and the formula for calculation of *E*_100_ using *C*_11_ and *C*_12_ at the pressure lower than 140 GPa.

## Abbreviations

BCC: Body-centered cubic
DAC: Diamond-anvil cell
DFT: Density functional theory
EAM: Embedded-atom method
EFS: Extended Finnis-Sinclair
EOS: Equation of state
FCC: Face-centered-cubic
LAMMPS: Large-scale atomic/molecular massively parallel simulator
MD: Molecular dynamics
NPT: Isothermal-isobaric ensemble
NVT: Canonical ensemble
OVITO: Open Visualization Tool
PBC: Periodic boundary conditions
Ta: Tantalum
