# Correction to: Temperature and Pressure Dependences of the Elastic Properties of Tantalum Single Crystals Under <100> Tensile Loading: A Molecular Dynamics Study

**DOI:** 10.1186/s11671-018-2576-4

**Published:** 2018-05-24

**Authors:** Wei-bing Li, Kang Li, Kang-qi Fan, Da-xing Zhang, Wei-dong Wang

**Affiliations:** 1 ZNDY of Ministerial Key Laboratory, Nanjing University of Science and Technology, Nanjing, 210094 People’s Republic of China; 2 McCormick School of Engineering and Applied Science, Northwestern University, Evanston, IL 60208 USA; 3 School of Mechano-Electronic Engineering, Xidian University, Xi’an, 710071 People’s Republic of China; 4 Research Center of Micro-Nano Systems, Xidian University, Xi’an, 710071 People’s Republic of China

## Correction

In the original publication of this article [1] the third author was typesetted by mistake. The correct name of the third author should be Kang-qi Fan. The publisher apologizes to the readers and authors for the inconvenience.

The original publication has been corrected.
